# Imaging of the appearance time of cerebral blood using [^15^O]H_2_O PET for the computation of correct CBF

**DOI:** 10.1186/2191-219X-3-41

**Published:** 2013-05-23

**Authors:** Nobuyuki Kudomi, Yukito Maeda, Yasuhiro Sasakawa, Toshihide Monden, Yuka Yamamoto, Nobuyuki Kawai, Hidehiro Iida, Yoshihiro Nishiyama

**Affiliations:** 1 Department of Medical Physics, Faculty of Medicine, Kagawa University, 1750-1 Ikenobe, Miki-cho, Kita-gun, Kagawa 761-0793, Japan; 2 Department of Clinical Radiology, Kagawa University Hospital, Kagawa 761-0793, Japan; 3 Department of Radiology, Faculty of Medicine, Kagawa University, Kagawa 761-0793, Japan; 4 Department of Neurological Surgery, Faculty of Medicine, Kagawa University, Kagawa 761-0793, Japan; 5 Department of Investigative Radiology, National Cerebral and Cardiovascular Center, Research Institute, Osaka 565-8565, Japan

**Keywords:** PET, CBF, Appearance time of blood, H_2_^15^O

## Abstract

**Background:**

Quantification of cerebral blood flow (CBF) is important for the understanding of normal and pathologic brain physiology. Positron emission tomography (PET) with H_2_^15^O (or C^15^O_2_) can quantify CBF and apply kinetic analyses, including autoradiography (ARG) and the basis function methods (BFM). These approaches, however, are sensitive to input function errors such as the appearance time of cerebral blood (ATB), known as the delay time. We estimated brain ATB in an image-based fashion to correct CBF by accounting for differences in computed CBF values using three different analyses: ARG and BFM with and without fixing the partition coefficient.

**Methods:**

Subject groups included those with no significant disorders, those with elevated cerebral blood volume, and those with reduced CBF. All subjects underwent PET examination, and CBF was estimated using the three analyses. The ATB was then computed from the differences of the obtained CBF values, and ATB-corrected CBF values were computed. ATB was also estimated for regions of interest (ROIs) of multiple cortical regions. The feasibility of the present method was tested in a simulation study.

**Results:**

There were no significant differences in the obtained ATB between the image- and ROI-based methods. Significantly later appearance was found in the cerebellum compared to other brain regions for all groups. In cortical regions where CBF was reduced due to occlusive lesions, the ATB was 0.2 ± 1.2 s, which was significantly delayed relative to the contralateral regions. A simulation study showed that the ATB-corrected CBF was less sensitive to errors in input function, and noise on the tissue curve did not enhance the degree of noise on ATB-corrected CBF image.

**Conclusions:**

This study demonstrates the potential utility of visualizing the ATB in the brain, enabling the determination of CBF with less sensitivity to error in input function.

## Background

Quantification of cerebral blood flow (CBF) is important for the understanding of normal and pathologic brain physiology. Parametric CBF images can be quantitatively measured using positron emission tomography (PET) with ^15^O-labeled water (H_2_^15^O) or carbon dioxide (C^15^O_2_), and several quantitative approaches have been developed to obtain CBF images for clinical assessment, such as the bolus administration of H_2_^15^O or C^15^O_2_ with autoradiography (ARG) [1,2] and dynamic protocols [3-5]. The ARG protocol applies the integration method from the bolus administration of C^15^O_2_ or H_2_^15^O [1,2], while the dynamic protocol applies optimization procedures such as the basis function method (BFM) [4], allowing the simultaneous estimation of multiple parameters, including uptake and washout rate constants of tracer and vascular volume.

Recently, Kudomi et al. developed a dual-tracer approach with the basis function method (DBFM) [6] for images of CBF as well as oxygen extraction fraction (OEF) and metabolic rate of oxygen (CMRO_2_). In the report, the authors demonstrated differences in sensitivity between the ARG and BFM computation algorithms due to error factors in input functions such as delay and dispersion. These errors resulted from the over- or underestimation of estimated parametric values, suggesting that the difference in the estimated parameters can be utilized as information regarding the degree of error in the error factors. Thus, the delay time in the input function, namely the appearance time of cerebral blood (ATB) (or tracer), may be estimated on a pixel-wise basis.

In this study, we estimated the ATB in brain regions using a pixel-wise approach based on the differences in estimated CBF values derived from the ARG and BFM using clinical C^15^O_2_ PET data. The feasibility of this method was tested by comparing the estimated ATB to that derived using a region-based analysis. On the basis of estimated ATB, CBF images were recomputed by shifting the time in tissue time-activity curves (TACs). The sensitivity of the ATB and resulting CBF, as well as the feasibility of our methodology, were tested in a simulation study.

## Methods

### Subjects

The subjects were the same as those described in our previous paper [7]. Briefly, subjects who had received PET examination for CBF, cerebral blood volume (CBV), OEF, and CMRO_2_ due to suspected cerebrovascular disorders were retrospectively selected from a clinical database in our hospital. We separated the subjects into three groups as follows: group 0, patients without any significant diagnosis or disorder (*n* = 10, 6 males and 4 females, weight 56.8 ± 12.0 kg, age 60.6 ± 18.7 years); group 1, patients with chronic stenosis or occlusion with elevated CBV (*n* = 9, 6 males and 3 females, weight 60.0 ± 14.1 kg, age 65.7 ± 14.7 years); and group 2, patients with chronic stenosis or occlusion with reduced CBF and elevated OEF (*n* = 10, 9 males and 1 female, weight 62.4 ± 11.1 kg, age 65.6 ± 12.4 years). All patients gave a written consent to participate in the study, which was approved by the University Ethical Committee.

### PET measurement protocol

The PET procedure was the same as that described in our previous paper [7]; however, only the PET scan data with C^15^O_2_ administration was used in this study. Briefly, all scanning was carried out in 2D mode using the Siemens ECAT HR^+^ scanner (Siemens-CTI, Knoxville, TN, USA). After transmission scans for 300 s, an emission scan was started with simultaneous C^15^O_2_ (1.5 GBq/min) inhalation for 2 min. The scan protocol consisted of 23 frames for a total of 600 s (1 × 30 s, 10 × 15 s, 10 × 30 s, and 2 × 60 s). During the C^15^O_2_ scan, blood was manually sampled (approximately 1 ml) through a catheter inserted in the right radial artery at 30, 45, 60, 75, 90, 105, 120, 135, 150, 165, 180, 210, 240, 270, 300, 360, 420, 480, 540, and 600 s from the PET start time. Radioactivity concentration in the blood samples was measured using an ARC400 well counter (Aloka, Mitaka, Tokyo, Japan).

### Data processing

Dynamic sinogram data were corrected for dead time in each frame in addition to detector normalization. Tomographic images were reconstructed from corrected sinogram data by the filtered back-projection method with a Hann filter. Attenuation correction was applied with transmission data. A reconstructed image consisted of a 128 × 128 × 63 matrix size with a pixel size of 1.7 × 1.7 mm and 2.4 mm with 23 frames.

Measured arterial blood TACs were calibrated to the PET scanner and then simultaneously corrected for delay and dispersion [8-10]. Specifically, the blood TAC was fitted with the four parameters of uptake rate (*K*_1_), washout rate (*k*_2_), delay time (Δ*t*), and dispersion constant to a whole-brain TAC extracted from a whole-brain region of interest (ROI). The corrected blood TAC was used as an input function. Parametric images of CBF were computed by the ARG [11,12] and by the BFM with the partition coefficient either fixed (=0.8 ml/g) (BFMF) [13] or non-fixed (BFMP) [4,7].

The ATB was defined as the time difference between the blood appearance of pixel location and the input function adjusted to the whole brain as above, where negative and positive times indicate earlier and later appearances, respectively. The ATB was estimated using the difference in estimated CBF values from two different computation methods, i.e., differences between ARG and BFMF and between BFMP and BFMF. We represented the difference as the ratio of two CBF values. In addition, the mean of two CBF values was also used to estimate ATB because the degree of the difference could depend on the CBF value. To compute the ATB, we used look-up tables matching the blood appearance time of pixel location as a function of the mean and the ratio of two CBF values. To create the table, multiple tissue curves were generated from the obtained input function, assuming CBF values ranging from 0.0 to 2.0 ml/min/g with a step of 0.01 ml/min/g. Then, CBF values were computed by ARG, BFMF, and BFMP using time-shifted input functions from −10 to 10 s with a step of 0.1 s. The shifted time corresponds to the ATB. Finally, two tables were created inputting mean and ratio of computed CBF values from the two sets of the two methods, i.e., ARG and BFMF, and BFMP and BFMF.

With regard to clinical data, the mean and ratio of CBF for ARG and BFMF and for BFMP and BFMF were calculated, and two appearance times were derived according to the tables obtained from the step described above. ATB was determined as the mean of the two derived appearance times. On the basis of the obtained ATB, the time in tissue TAC was shifted pixel-wise in the dynamic image, and the CBF image with ATB correction was computed using BFMF (BFMAT).

Regional appearance time was also estimated by fitting the input function with three parameters, *K*_1_, *k*_2_, and Δ*t*, to regional brain TACs. The TACs were obtained from circular ROIs placed on summed images in the frontal, temporal, parietal, occipital, deep gray matter, and cerebellar cortical regions in each hemisphere for all subjects. The diameter of each ROI was 12.1 mm.

### Data analysis

The ATB and CBF values determined by ARG, BFMF, BFMP, and BFMAT were extracted for all ROIs; and the means and SDs were obtained across all subjects in each group. The ATB values calculated using the pixel-wise and ROI-based methods were compared using Student's *t* test. In addition, in group 2, ATB values in regions diagnosed with disease were compared to those in the corresponding regions in the contralateral hemisphere by the *t* test. *P* < 0.05 was considered statistically significant.

### Error analyses in simulation

Characteristics of the present procedure, namely how CBF and the ATB values could be correctly estimated when input appeared earlier or delayed, were tested in a simulation. Error propagation to the ATB and CBF was evaluated for the following three error sources: first, error in estimated delay time in the input function [8]; second, error in dispersion correction in the input function [9], by shifting the time constant from −5 to 5 s to generate tissue TACs as below, where a negative error represents undercorrection, as described previously [6,14,15]; and third, error in the assumed blood/tissue partition coefficient, *p* (=0.8 ml/g) [6,13,14], by varying *p* from 0.7 to 0.9 ml/g to generate tissue TACs.

For the simulation, an input function obtained in the present clinical study was defined as true. Applying the single tissue compartment model as the kinetic formula for water [1], tissue TACs were generated for ‘normal’ (CBF = 0.50 ml/g/min), ‘ischemic’ (CBF = 0.30 ml/min/g), and ‘hyperperfusion’ (CBF = 0.70 ml/min/g) conditions. To simulate the delay effect, the CBF and ATB values were computed using the generated tissue TACs by shifting the time of the input function from −5 to 5 seconds, where a negative time represents an earlier appearance of the input function. To simulate the effects of the dispersion time constant and partition coefficient, CBF and ATB values were then calculated by assuming *p* = 0.8 ml/g, using the true input function as well as ±5-s time-shifted input functions and the TACs generated by varying the dispersion time constant or partition coefficient. Errors in the calculated CBF and ATB values were plotted as a function of differences in the assumed delay time, dispersion time constant, and the partition coefficient.

The influence of noise on the accuracy of the estimated ATB as well as on the corrected CBF by the BFMAT was explored. First, to this purpose, we used the above true input function and the generated tissue TACs. Then, tissue TACs with noise were generated by imposing Gaussian noise with levels of 15% at peak and 15% of the square root of counts at the other points. This procedure was repeated 10,000 times for each of the assumed normal, ischemic, and hyperperfusion conditions, yielding three sets of 10,000 noisy tissue TACs. The framing of generated tissue TACs was the same as that of the clinical PET data. Then, CBF and ATB values were computed using the time-shifted input function from −5 to 5 s and the generated noisy tissue TACs. Mean and SD of calculated ATB and CBF were obtained.

## Results

### Experiments

The present ATB and CBF computation program successfully calculated the parametric images for PET data on all subjects. The computation time for parametric images was approximately 85 s using a standard PC installed with GNU/Linux (fc16.x86_64 64 bit; CPU, Intel Core i7 3.07 GHz; memory, 16 GB).

**Figure 1:**
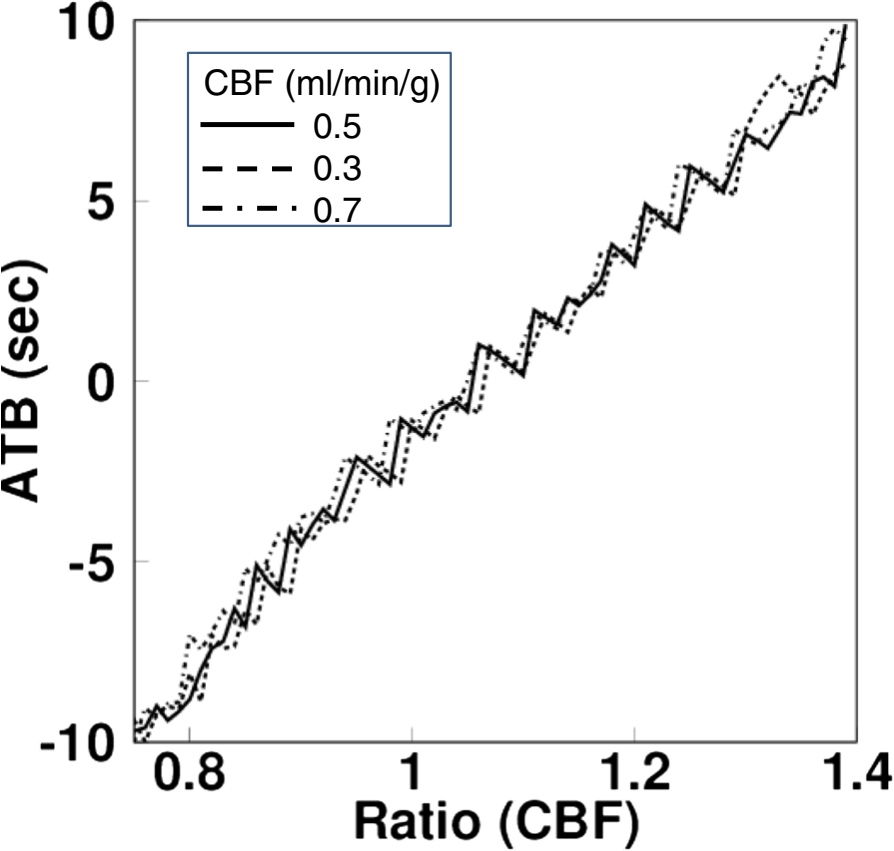
**An example of a look-up table.** Obtained from one of the present subjects showing the function of the CBF ratio between ARG and BFMF for the CBF mean value of 0.5, 0.3, and 0.7 ml/min/g, respectively.

A look-up table obtained from one of the present subject is shown in Figure 1 as a function of the CBF ratio between ARG and BFMF for the CBF mean value of 0.5, 0.3, and 0.7 ml/min/g, respectively. There appeared to be the same mean and ratio of CBF values with different ATB values, but those ATB values were similar. In that case, the mean of those different ATB values were inputted into the look-up table.

The mean and SD of ATB values for all ROIs were 0.0 ± 2.5 and 0.1 ± 3.0 s for group 0 as derived by the image- and ROI-based methods, respectively. Those were 0.0 ± 2.6 and 0.0 ± 3.2 s, respectively, for group 1 and 0.0 ± 2.4 and 0.1 ± 2.5 s, respectively, for group 2. There were no significant differences in estimated ATB values between the image-based and ROI-based methods in any group. The means and SD of the ATB and CBF values derived by ARG, BFM, BFMP, and BFMAT for each ROI are summarized in Table 1. Significantly later appearance was found in the cerebellum compared to the other regions for all groups. In group 2, the ATB value in CBF-reduced regions was 0.2 ± 1.2 s, which was significantly delayed compared to the ATB value of −0.6 ± 1.3 s in the corresponding contralateral hemisphere regions. The mean and SD of the partition coefficient for all ROIs was 0.80 ± 0.07 and 0.80 ± 0.06 ml/g for group 0, as calculated by BFMP and BFMAT, respectively. Those were 0.82 ± 0.07 and 0.82 ± 0.06 ml/g, respectively, for group 1 and 0.80 ± 0.07 and 0.80 ± 0.06 ml/g, respectively, for group 2. There were no significant differences between BFMP and BFMAT in the estimated partition coefficient for the parietal, frontal, temporal, deep gray, and occipital regions in any of the groups. However, in the cerebellum, a significant difference was obtained between BFMP and BFMAT, and the partition coefficients for each method were 0.88 ± 0.05 and 0.87 ± 0.04 ml/g in group 0, 0.88 ± 0.03 and 0.87 ± 0.02 ml/g in group 1, and 0.87 ± 0.05 and 0.85 ± 0.04 ml/g in group 2. In group 2, the partition coefficient values computed by BFMP and BFMAT in CBF-reduced regions were 0.80 ± 0.04 and 0.80 ± 0.06 ml/g, respectively, which were not significantly different.

**Table 1:** Means and SDs of ATB and CBF obtained using the four methods for groups 0, 1, and 2

	**Parietal**	**Frontal**	**Temporal**	**Occipital**	**Deep gray matter**	**Cerebellum**
Group 0						
ATB (s)	−0.6 ± 1.6	−0.3 ± 1.3	−0.4 ± 1.4	0.1 ± 1.7	−0.8 ± 1.7	1.9 ± 1.3^a^
CBF (ml/min/g)						
BFMAT	0.36 ± 0.06	0.38 ± 0.09	0.42 ± 0.09	0.45 ± 0.12	0.41 ± 0.12	0.45 ± 0.11
ARG	0.35 ± 0.06	0.37 ± 0.07	0.41 ± 0.08	0.45 ± 0.09	0.40 ± 0.10	0.46 ± 0.09
BFMF	0.37 ± 0.06	0.39 ± 0.08	0.42 ± 0.08	0.46 ± 0.09	0.42 ± 0.11	0.44 ± 0.09
BFMP	0.37 ± 0.06	0.39 ± 0.08	0.42 ± 0.10	0.46 ± 0.09	0.42 ± 0.10	0.48 ± 0.09
Group 1						
ATB (s)	−1.1 ± 1.7	−0.1 ± 1.9	−1.1 ± 1.8	0.6 ± 1.7	−0.5 ± 1.8	2.2 ± 2.0^a^
CBF (ml/min/g)						
BFMAT	0.33 ± 0.09	0.36 ± 0.13	0.39 ± 0.13	0.43 ± 0.07	0.43 ± 0.09	0.46 ± 0.16
ARG	0.32 ± 0.08	0.36 ± 0.11	0.38 ± 0.12	0.43 ± 0.07	0.42 ± 0.09	0.45 ± 0.12
BFMF	0.33 ± 0.09	0.36 ± 0.11	0.39 ± 0.10	0.43 ± 0.06	0.44 ± 0.08	0.43 ± 0.10
BFMP	0.33 ± 0.09	0.37 ± 0.11	0.38 ± 0.12	0.43 ± 0.07	0.44 ± 0.09	0.47 ± 0.13
Group 2						
ATB (s)	−0.8 ± 1.0	0.3 ± 1.4	−0.5 ± 1.0	−0.2 ± 1.5	−0.4 ± 1.7	1.6 ± 1.7^a^
CBF (ml/min/g)						
BFMAT	0.32 ± 0.07	0.34 ± 0.10	0.36 ± 0.11	0.38 ± 0.10	0.38 ± 0.11	0.39 ± 0.14
ARG	0.31 ± 0.07	0.34 ± 0.10	0.36 ± 0.11	0.38 ± 0.09	0.37 ± 0.10	0.40 ± 0.10
BFMF	0.33 ± 0.07	0.34 ± 0.09	0.37 ± 0.10	0.38 ± 0.10	0.38 ± 0.10	0.38 ± 0.08
BFMP	0.33 ± 0.07	0.35 ± 0.10	0.37 ± 0.11	0.39 ± 0.09	0.38 ± 0.10	0.43 ± 0.11

ATB, appearance time of blood (negative number corresponds to earlier appearance); CBF, cerebral blood flow. ^a^Significantly different.

**Figure 2:**
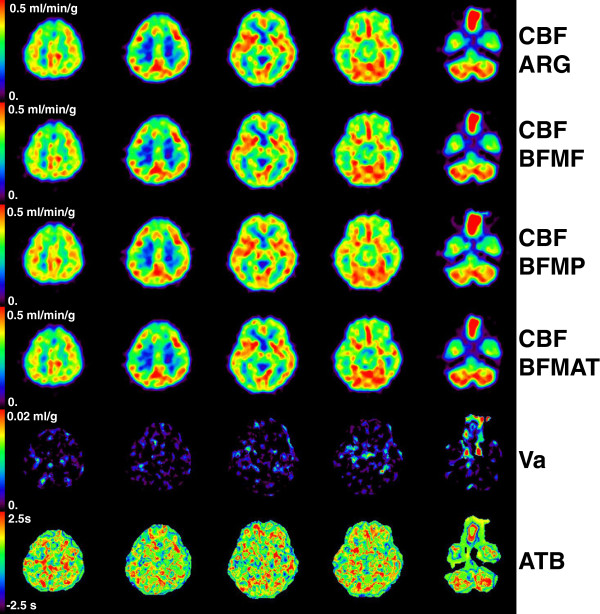
**Representative view for group 0.** Shown are the cerebral blood flow (CBF) obtained by ARG, BFMF, BFMP, and ATB correction, cerebral arterial blood volume (Va), and the appearance time of blood (ATB) images for a subject in group 0.

**Figure 3:**
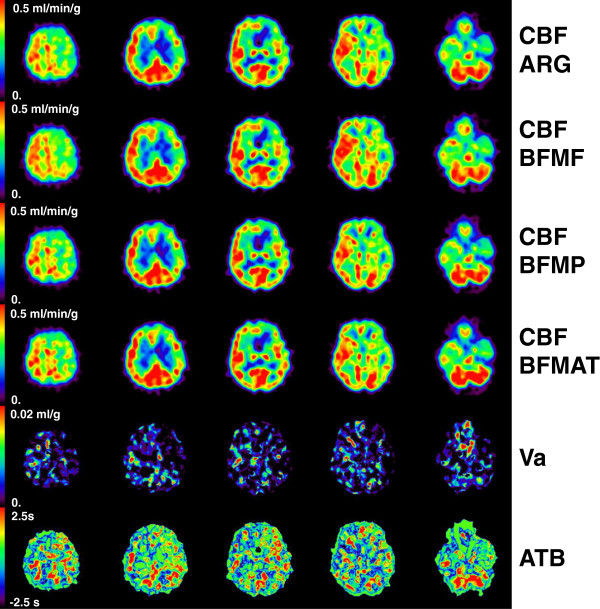
**Representative view for group 2.** Shown are the cerebral blood flow (CBF) obtained by ARG, BFMF, BFMP, and ATB correction, cerebral arterial blood volume (Va), and the appearance time of blood (ATB) images for a subject in group 2.

Representative CBF images derived using all four methods as well as generated ATB images are shown in Figures 2 and 3 for groups 0 and 2, respectively. The quality of CBF images derived by BFMAT methods was not deteriorated compared to those derived by the other methods. In the temporal region in the left hemisphere (appearing on the right side) in Figure 2, CBF seems to be lower in BFMF than in ARG and BFMP, and in the corresponding region, ATB indicates a later appearance. In contrast, in the frontal region in the right (contralateral) hemisphere in Figure 3, CBF seems to be higher in BFMF than in ARG and BFMP, and in the corresponding region, ATB indicates an earlier appearance. The CBF derived by the BFMAT seems accordingly corrected. In Figure 3, the ATB seems to be delayed in regions with reduced CBF compared to the same regions in the contralateral hemisphere.

### Error analyses

**Figure 4:**
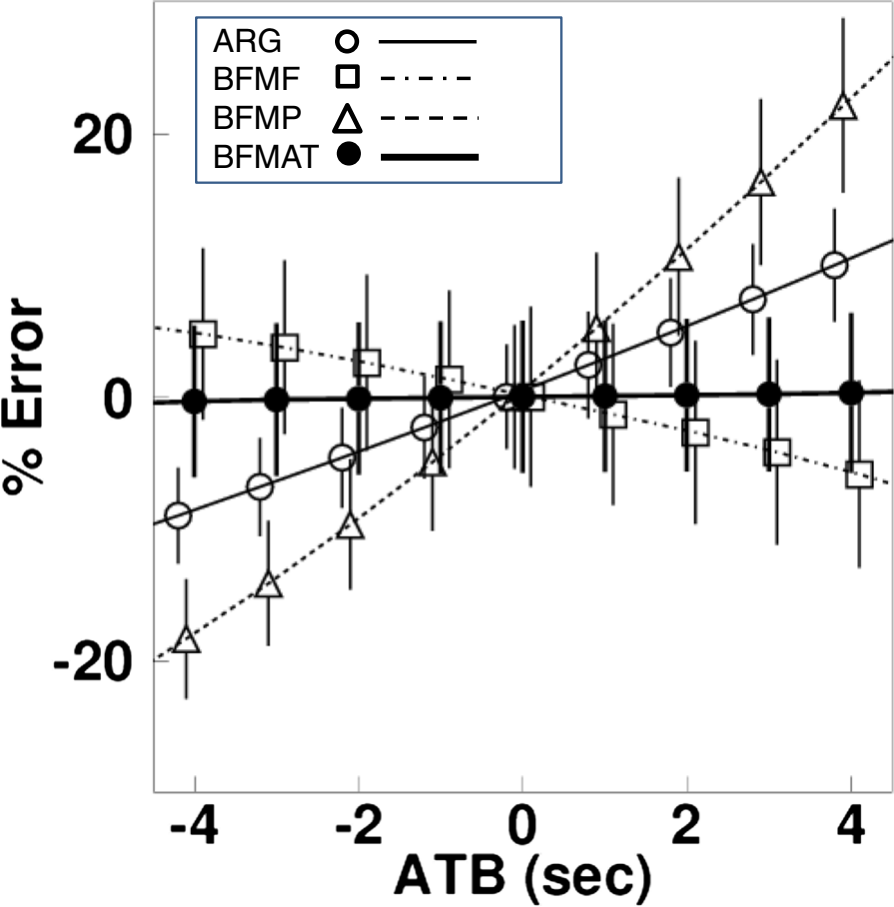
**Sensitivities of CBF to the appearance time error.** Results of the simulation study demonstrating the effects of different appearance time of the arterial input function on the cerebral blood flow (lines) and on the calculated mean (plots) and SD (error bars) of CBF from the noisy TAC. The assumed CBF value was 0.5 ml/min/g. Positive and negative values of errors in delay time indicate earlier and later appearance, respectively. Results are plotted for the autoradiography (ARG), the basis function methods with fixing partition coefficient (BFMF), the basis function methods without fixing partition coefficient (BFMP), and ATB correction (BFMAT).

Sensitivities of CBF to the appearance time error for the normal condition are shown in Figure 4. The degrees differ depending on the computation algorithms; in particular, BFMF had opposite tendencies to ARG and BFMP toward over- and underestimation for positive and negative delay times. CBF was well estimated by BFMAT, namely it was identical to the assumed values. For the ischemic and hyperperfusion conditions, the errors in CBF were also negligible after ATB correction, and the ATB was identical to the assumed shift time. For the effect of noise in tissue TAC on CBF, the mean and SD are shown in Figure 4 for the normal condition. The sizes of the SD for the normal condition were identical regardless of the appearance time and were 4.0% for ARG, 5.6% for BFMF, 7% for BFMP, and 5.6% for BFMAT, indicating that the size of CBF image noise was not enhanced between BFMF and BFMAT. For the ischemic condition, the SDs were 3.9% for ARG, 6.2% for BFMF, 6.7% for BFMP, and 6.1% for BFMAT; and for the hyperperfusion condition, the SDs were 4.5% for ARG, 5.2% for BFMF, 7.0% for BFMP, and 5.2% for BFMAT. These were independent of the appearance time. The effect of noise in tissue TAC on ATB, i.e., mean on the estimated ATB, was identical to the assumed ATB, and SD was 1.7 s independent of appearance time. For the ischemic and hyperperfusion conditions, the means were also identical to the assumed ATB, and SDs were 2.1 and 1.6 s, respectively.

**Figure 5:**
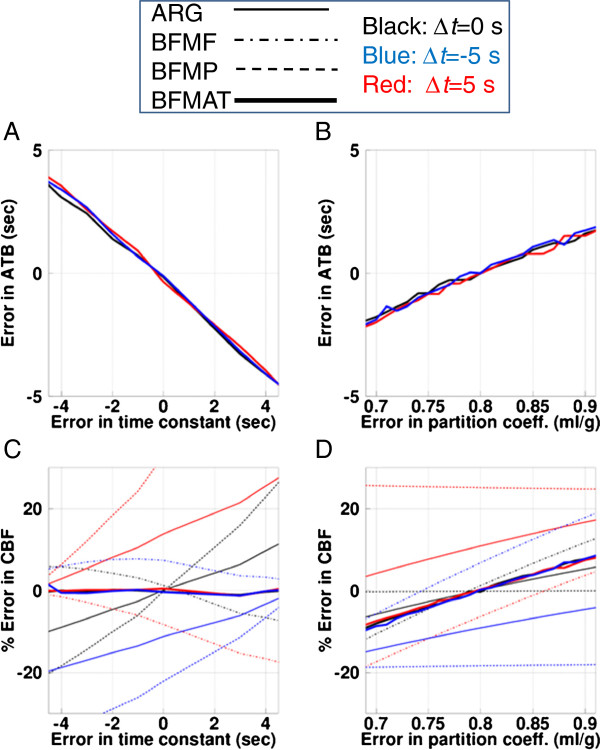
**Sensitivities of ATB and CBF to errors in dispersion time constant and partition coefficient value.** Results of the simulation study demonstrating the effects of errors of the assumed dispersion time constant of the arterial input function (**A**, **C**) and errors in the assumed partition coefficient (*p*, ml/g) (**B**, **D**) on the calculated appearance time of blood (ATB) (**A**, **B**) and the calculated cerebral blood flow (CBF) (**C**, **D**). Positive and negative values of errors in the time constant indicate undercorrection and overcorrection of dispersion time, respectively. Results are plotted for the autoradiography (ARG), the basis function methods with fixing partition coefficient (BFMF), the basis function methods without fixing partition coefficient (BFMP), and ATB correction (BFMAT). Δ*t*, shifted time of appearance time of input.

Sensitivities of ATB and CBF to errors in the dispersion time constant and the partition coefficient value for the normal condition are shown in Figure 5. The magnitude of error in the ATB was ±2 s for ±2-s error in the dispersion time constant (Figure 5A), independent of the time shift of the input function, and was ±1.4 s for the assumed partition coefficient of 0.75 and 0.85 ml/g (Figure 5B). The magnitude of error in the CBF was negligible for errors in the dispersion time constant when using BFMAT, i.e., after ATB correction, although the magnitude was dependent on the time constant when computed by the other methods (Figure 5C). The error magnitude in the CBF computed by BFMAT was ±4% for 0.75 and 0.85 ml/g for the partition coefficient, independent of the time shift in the input function, which was smaller than that for BFMF and the same as that for the ARG (Figure 5D).

The error propagation in the ABT and CBF from the dispersion time constant computed by BFMAT was identical under both the ischemic and hyperperfusion conditions. The error propagation from the partition coefficient was found to be decreased in the ischemic condition and increased in the hyperperfusion condition.

## Discussion

In the present study, we estimated the ATB in the brain in an image-based fashion, from the sensitivity differences of different computation methods, including the ARG, BFMF, and BFMP. On the basis of the estimated ATB, CBF values were computed using the BFMAT by shifting the time in tissue TACs. The ATB obtained in image-based analysis was not significantly different from that obtained by ROI-based analysis. Significantly later appearance was found in the cerebellum compared to the other regions in all three groups. In the regions where CBF was reduced due to occlusive lesions, the estimated ATB was 0.2 ± 1.2 s, which was significantly delayed compared to the value of −0.6 ± 1.3 s in the unaffected contralateral regions. In our simulation study, estimated CBF was insensitive to errors in appearance time and dispersion time constant. With regard to the variation of the partition coefficient, systematic errors in CBF persisted after ATB correction. However, the degree of error was smaller than that computed by BFMF and was the same as that computed by ARG. As a whole, this study suggests a method for visualizing the ATB in the brain and correcting CBF that is less sensitive to the error sources in PET examination.

The computation of CBF using bolus inhalation of C^15^O_2_ or injection of H_2_^15^O generally requires the determination of the common zero time between the measured arterial blood and brain tissue curves in every study using appropriate procedures [8]. However, the accuracy of computed regional CBF is still affected by regional differences in the ATB. For further correction for regional time differences, the present study used an image-based approach to estimate the ATB. None of the ATB values obtained from images of the parietal, frontal, temporal, deep gray matter, occipital, or cerebellar cortical regions were significantly different from those derived using the ROI-based analysis. The ATB for different brain regions varied from −1.0 to 2.0 s from the zero times obtained from whole-brain TACs (Table 1) in group 0, 1, and 2 subjects. The ATB in the cerebellar cortical region varied from around 1.5 to 2.0 s and was significantly delayed compared to that in the other regions. A previous study has demonstrated similar time differences of around ±2-s variation and 2-s delay in the cerebellum [8]. In anatomical terms, vascular supply patterns are basically subject to individual variation, and it may not be possible to define universal ATB patterns in the brain. However, there are still frequent and dominant patterns identifiable in the human brain [16]. The finding of no significant differences in the ATB in the parietal and frontal regions (territory of the terminal branches of the anterior cerebral artery (ACA)), the temporal region (territory of the middle cerebral artery (MCA)), the occipital (territory of the posterior cerebral artery (PCA)), and the deep gray (the territory of penetrating branches of the ACA, MCA, and PCA), all supplied from the internal carotid artery, may suggest a similar degree of blood transit time supply. However, the significant difference in ATB in the cerebellar region, which is the territory of the posterior inferior cerebral artery through the vertebral artery, suggests that the transit time could be longer.

In the regions with reduced CBF, i.e., those affected by occlusive lesions, the ATB was significantly delayed, and that delay can be clearly seen in the ATB image (Figure 3). These findings suggest that the ATB measure could be of interest for identifying occluded areas or as a pathophysiological indicator in patients with occlusive lesions. It may be of interest to compare the ATB with the OEF in the occlusive area. The present study focused on ATB and CBF, so only the C^15^O_2_ PET scan and data analysis procedures were presented. Since the PET protocol was originally designed to obtain CBF, CBV, OEF, and CMRO_2_[8], it was possible to compare the ATB and OEF. We determined the correlation between these two measures and found that it was slight, i.e., *r* = 0.25 for all ROIs drawn in group 2 subjects and *r* = 0.45 for CBF-reduced regions (data not shown). This slight correlation might be due to the structure of the cerebral circulation system; specifically, variation in the ATB due to cerebral location may obscure the delayed appearance in the occlusive areas. Further study to determine the possibility of ATB as a clinical indicator, taking into account the cerebral circulation structure, is warranted.

The present experimental study showed that the regional variation in the ATB was around ±1.5 s, which caused errors in computed CBF values that depended on the computation method used (Figure 4). As the present simulation showed, the difference of ±1.5 s led to errors of 3%, 3%, and 7% in CBF computed by ARG, BFMF, and BFMP, respectively (Figure 5C). To correct for the error in delay and reduce the degree of error in the CBF value, the present method was used to determine the pixel-wise ATB within a reasonable computation time of approximately 85 s. While determining the ATB, the accuracy could be affected by framing of PET data and by noise in tissue TAC. The present simulation, assuming the same framing condition as the clinical data, showed that the mean ATB reproduced the shift time, and the SD was 1.7 s. This relatively improved temporal resolution was achieved because the ATB was estimated from difference in CBF values, which is dependent on the computation method and independent of the framing, particularly with the ARG. We applied ROI-based analysis to the noisy TACs after summing 50 of the noise-added TACs. The estimated SD of ATB was 2.5 s, suggesting that the present pixel-wise method, depending on CBF accuracy, may provide more accurate shift time than the ROI-based method in the present clinical setup.

Additionally, the estimated mean CBF value was identical to the assumed CBF value, independent of shift time; and particularly, the magnitude of noise on CBF was not enhanced in comparison to the method without ATB correction, i.e., BFMF. The noise on CBF was not enhanced by either ARG or BFMP with ATB correction (data not shown). The lesser enhancement of noise in CBF following ATB correction might be due to the three CBF values competing with each other as additional information and thus decreasing noise, although ATB correction enhanced noise to the same degree.

The CBF value was affected by errors in the applied dispersion time constant in the input function. The simulation performed here revealed that the estimated ATB was affected by the error in the dispersion time constant; however, the error in the estimated CBF after correction was negligible compared to the error in both the time constant and the appearance time (Figure 5C). The CBF and ATB values were also affected by errors in the partition coefficient. For the error in the partition coefficient, both the ATB and CBF were affected when calculated by BFMAT; however, the degree of error in CBF was smaller than that computed by BFMF and the same as that by ARG (Figure 5D). The partition coefficient obtained using BFMP and that obtained after ATB correction were around 0.80 ml/g which did not change significantly with ATB correction, except for the cerebellar region, and was identical to the fixed value for ARG, BFMF, and BFMAT. The significant difference in the cerebellar region could be partly derived from the significant delay of the appearance time, although the obtained appearance time was identical between the image-based and ROI-based analyses. The computed CBF value after ATB correction was identical among ARG, BFMF, and BFMP; and to determine the final CBF value, the present study applied BFMF because this method was less affected by noise than BFMP, was useful in determining arterial blood volume (Va), and did not require the partition coefficient. When the partition coefficient is required, BFMP with ATB correction is available.

A previous approach suggested around ±2 s of regional variation in the appearance time of the tracer [8]. For dispersion, a corresponding difference in the time constant (Δ*τ*) could be roughly estimated as Δ*τ* = ±0.6 s for ±2-s appearance time difference if we apply the relationship between the time difference (Δ*t*) and the constant as Δ*τ* = 0.31Δ*t* − 0.30 [17]. Additionally, the effect might be smaller in the cerebral capillary circulation system since dispersion is assumed to be caused by laminar flow [18], and the degree of dispersion in small vessel would therefore be small. Thus, we presume that the effect of variation in appearance time is larger than that of dispersion, and on the basis of this, we developed the computation strategy for ATB. The estimated CBF computed using BFMAT, which is important for clinical diagnosis and research purposes, was less sensitive to variations in both appearance time and dispersion time constant, as demonstrated by the simulation we performed.

In a previous report, the authors demonstrated differences in sensitivity between the ARG and BFM computation algorithms due to delay in input functions [6]. In the present study, ATB was estimated using the difference in estimated CBF values from the different computation methods, i.e., differences between ARG and BFMF and between BFMP and BFMF. For practical estimation, we represented the difference as the ratio of two CBF values. Additionally, it was not guaranteed that the degree of error in CBF was independent of the CBF value, although the look-up tables generated showed similar ATB values independent of CBF values (Figure 1). Thus, we created the tables as a function of the mean and ratio of the estimated CBF values. In our preliminary assessment, the look-up table created for estimation was dependent on the input function applied. Thus, a table was created for every data computation applied. In creating the table, the means and ratios of CBF values were obtained by shifting the times of the input function applied. The shifted time corresponds to the ATB. There could be multiple ATB values as an element on a table, or there could be no data. In the former case, the mean of multiple values was inputted as the element. In the latter case, the interpolation of adjacent elements was inputted. In both cases, accuracy could be deteriorated, and this may have appeared as a fluctuation of the table value (Figure 1) and thus of the estimated ATB (Figures 4 and 5A,B). However, the degree of fluctuation does not seem to be large.

The method used in the current study generated arterial blood volume images after ATB correction. The Va contains only the arterial blood volume rather than the total arterial and venous blood volume that can be determined using a C^15^O PET scan [6]. For Va images derived using the BFMF formula without ATB correction, qualitative and quantitative accuracy suffered from noise and from further systematic error in the measured and corrected input functions. Specifically, the error magnitude of the Va value, propagated from errors in the input function such as delay and dispersion corrections, was quite large [19]. The present algorithm allows for the correction of regional variation in the ATB based on the estimated ATB and, thus, may reduce the systematic error in the estimated Va value, although the estimated Va could still be affected by dispersion in input function. The procedure can be extended to another approach for the DBFM method [6], for additional OEF, CMRO_2_, and *V*_0_ images, where the *V*_0_ contains both the arterial and venous blood volume. Further studies should be carried out to evaluate the significance of the Va and *V*_0_ parameters by applying the present procedure.

The present simulation study revealed that the computed CBF after ATB correction was less sensitive to errors in the input function, such as delay and dispersion. The errors in CBF due to these delay and dispersion factors may also reduce accuracy when obtaining image-derived input functions [20] because errors in both measured and image-derived input lead to differences in CBF values. The present CBF computing procedure avoids such effects and, thus, may contribute to the further development of methods for image-derived input.

## Conclusions

In conclusion, this study suggests the possibility of visualizing the ATB in the brain and correcting CBF so that it is less sensitive to the error sources inherent in PET examination.

## Competing interests

The authors declare that they have no competing interests.

## Authors' contributions

All authors contributed substantially to the scientific process leading to this manuscript. NKu, YM, YY, NKa, and YN participated in the study design and the data analysis. NKu and HI performed the image analysis and simulation. YM, YS, and TM acquired data on the subjects. NKu drafted the manuscript. All authors read and approved the final manuscript.
